# Mining data from hemodynamic simulations via Bayesian emulation

**DOI:** 10.1186/1475-925X-6-47

**Published:** 2007-12-13

**Authors:** Vijaya B Kolachalama, Neil W Bressloff, Prasanth B Nair

**Affiliations:** 1 Biomedical Engineering Center, Harvard-MIT Division of Health Sciences and Technology, Cambridge, MA, 02139, USA; 2 Computational Engineering and Design Centre, School of Engineering Sciences, University of Southampton, SO17 1BJ, UK

## Abstract

**Background:**

Arterial geometry variability is inevitable both within and across individuals. To ensure realistic prediction of cardiovascular flows, there is a need for efficient numerical methods that can systematically account for geometric uncertainty.

**Methods and results:**

A statistical framework based on Bayesian Gaussian process modeling was proposed for mining data generated from computer simulations. The proposed approach was applied to analyze the influence of geometric parameters on hemodynamics in the human carotid artery bifurcation. A parametric model in conjunction with a design of computer experiments strategy was used for generating a set of observational data that contains the maximum wall shear stress values for a range of probable arterial geometries. The dataset was mined via a Bayesian Gaussian process emulator to estimate: (a) the influence of key parameters on the output via sensitivity analysis, (b) uncertainty in output as a function of uncertainty in input, and (c) which settings of the input parameters result in maximum and minimum values of the output. Finally, potential diagnostic indicators were proposed that can be used to aid the assessment of stroke risk for a given patient's geometry.

## Introduction

Vascular diseases such as atherosclerosis and thrombosis are known to cause fluid mechanic derangements that affect blood flow in arteries [1]. Previous research involving *in vitro *studies [2-5] aimed at understanding these disorders have found that the main factors contributing to changes in blood flow patterns are the pulsatile behavior, viscosity of blood, the geometry of arteries and the arterial wall distensibility [1,6]. Of these, geometry of the vessel was found to be the most important factor influencing the flow behavior [6]. In recent years, *in silico *blood flow simulations have gained lot of popularity. In particular, three dimensional computational fluid dynamics (CFD) studies at the sites of curvature, bifurcations, and junctions have facilitated the identification of vulnerable atherogenic sites [1,7,8]. Simulations were performed to analyze blood flow patterns at several sites such as carotid [9-13], coronary [14-17], aortic [18,19] and iliac [20] arteries. Many of these have highlighted the disturbed flow patterns caused due to alterations in arterial geometry. Furthermore, fluid mechanical forces such as wall shear stress (WSS) have been identified to play a major role in the pathogenesis and pathophysiology of atherosclerosis [21].

Most of the work in the literature on computational hemodynamics assume that arterial geometry definition is precisely known. In practice, however, it is known to vary both with and within individuals [22,23]. Hence, there is a need for efficient numerical methods that can systematically account for geometric uncertainty and predict true flow behavior in real time. This problem is particularly challenging because it is difficult to characterize the observed geometric variability using a small number of variables – straightforward univariate or bivariate parameter studies that involve varying a subset of the geometric variables tend to be of limited use. A statistical assessment hence becomes beneficial to gain insights into the relationship between flow patterns and geometric attributes. The basic idea of this approach is to construct probabilistic models for the input uncertainties and subsequently propagate this through the computer model to assess the impact of variability in inputs on the outputs of interest. A standard approach for statistical analysis is the Monte Carlo simulation technique [24], where the computer model is run repeatedly for randomly generated values of the inputs, and subsequently, the resulting data is postprocessed to estimate the output statistics. However, due to the requirement of a large sample-size, this approach becomes computationally prohibitive, particularly when high-fidelity models are used.

In the present work, we proposed the application of Bayesian Gaussian process modeling [25-27] to study the relationship between geometric factors and hemodynamic metrics. The present approach can be viewed as a computer-based data mining strategy which extracts useful information and synthesizes interesting relationships from datasets generated during multivariable parameter studies. The input and output observational data was generated by running computer simulations on selected cases and emulators constructed via Bayesian Gaussian process modeling were used to analyze and summarize the data in novel ways that are understandable and can potentially be of clinical benefit.

To demonstrate the applicability of the proposed approach, the human carotid artery bifurcation was chosen as the anatomical site for analysis since it is a common site for arterial disease to occur [6]. A novel three dimensional parametric computer aided design (CAD) representation of the human carotid artery was defined. The objective behind using a parametric geometry model for statistical analysis is so that a range of probable geometries can be automatically generated. However, comparison between the data obtained for different simulations requires a method for ranking individual performance. Whilst it is possible to visually differentiate surface contours and/or velocity profiles between any two cases, a numerical value (metric or indicator) is preferable because it eases the level of comparison. A range of metrics are available in the literature, and most of these evaluate shear stress related expressions on a cell by cell basis [3,5,28-34]. Following some of this work, we considered the maximal wall shear stress (MWSS) as the metric for statistical analysis. It has already been shown that large changes in the magnitude of MWSS can play a role in the embolic mechanism by which carotid lesions can induce stroke [30]. Hence, it is important to understand the correlation between the geometric variability and MWSS in the human carotid artery.

A design of experiments (DOE) approach [35] was employed to generate a set of candidate geometries for steady state three dimensional flow analysis. The data generated from these runs was then used to construct a Bayesian Gaussian process emulator which approximated the MWSS as a function of the geometric variables. This model was subsequently employed as a computationally cheap emulator to compute the statistics of the output when the inputs were modeled as random variables and to estimate the degree of influence of each geometry parameter on the MWSS. Later, the application of the Bayesian emulator to identify geometries that have the highest and lowest MWSS values was demonstrated. Subsequently, pulsatile simulations on the same candidate geometries were performed to compare these results with the steady state case. Finally, we proposed potential diagnostic indicators that are capable of estimating the degree of severity with respect to the MWSS metric for a given patient's geometry thereby aiding the assessment of stroke risk.

## Methods

### Parametric model of the carotid bifurcation

Parametric computer aided design (CAD) definition of the carotid bifurcation enables the automatic generation of typical geometries by varying the parameters in the baseline CAD model. In so doing, not only is it possible to explore the impact of alternative definitions on the flow and its associated shear stress parameters but also, more importantly, the relationships between hemodynamics and a wide range of geometric parameters can be investigated in detail. The power of parametric geometry representation lies in its ability to simply generate a range of alternative geometries using the baseline shape as a template that is then reconfigured according to new values of the variable parameters. Note that it is also possible to morph the parametric model in order to reproduce a patient-specific geometry obtained from magnetic resonance imaging (MRI) [36-38] or contrast-enhanced x-ray computer tomography [39] scans. Image reconstruction techniques have reached a new level in the recent past where realistic geometry shapes were extracted from these scans in real time and subsequently, CFD studies were performed. In the context of our work, for example, the CAD model parameters can be estimated from a scan by solving an optimization problem involving minimization of an appropriate distance metric characterizing how well the CAD model reproduces the scanned geometry. In combination with regularization methods, the parametric CAD approach can be potentially useful in smoothening geometries obtained from imaging techniques.

Careful consideration of a number of geometry descriptions including [6] and [40] shows that, from a CAD perspective, insufficient information is available in all of them to construct a parametric CAD model from scratch, without making a number of assumptions, and/or unrealistic constraints are imposed that limit the overall flexibility of the models. Consequently, new parametric CAD definitions of the carotid geometry were presented in this paper. There are some similarities with older models but a number of important innovations were introduced that yield complete and reusable definitions and provide sufficient flexibility to facilitate parametric studies. This CAD representation was mainly based on the Y-shaped model developed in [6].

Appendix A describes the construction of the CAD model that is designed to provide a more complete representation (than previously available) of the human carotid artery geometry. Table 1 summarizes the values of all the parameters used in the baseline geometry. To construct a complete parametric geometry, a CATIA [41] macro was run each time a new model was required. This macro used parameters contained within a text file. The three dimensional parametric CAD model of the human carotid artery bifurcation thus generated was exported for mesh generation using GAMBIT [42]. All the meshes were constructed from scratch in response to a template journal file (which includes all the batch commands) that was read by GAMBIT [42]. In this way, the journal file was used for facilitating automatic mesh generation and subsequently, the mesh file was exported for CFD simulations using FLUENT [43].

**Table 1:** Dimensions of the human carotid artery bifurcation (Locations in Figure 1). Note that the split ratio values are not mentioned here and units of all the parameters shown here (excluding the angles) are in mm

*Description*	*Location*	*Mean value*
Angles	1	25.1°
	2	25.4°

External carotid	3	5.6
	4	15.0
	5	5.5
	6	15.0
	7	4.6
	8	15.0
	9	4.6

Internal carotid	10	8.3
	11	7.28
	12	8.9
	13	7.2
	14	8.2
	15	9.52
	16	6.0
	17	12.0
	18	5.7
	19	9.0
	20	5.7

Common carotid	21	8.0
	22	24.0
	23	8.0
	24	24.0
	25	8.0
	26	24.0
	27	8.0

Other	28	8.0

### Bayesian Gaussian process modeling

In this section, we describe the theoretical and computational aspects of Bayesian Gaussian process modeling and describe its application to mine data obtained from computational simulations. To illustrate, consider a computer code which takes as input the vector **x **∈ ℝ^*p *^and returns a scalar output *y*(**x**). Let a design of computer experiments (DOE) strategy be applied to decide the settings of the inputs at which the computer code must be run [35]. In the context of hemodynamics, this step essentially involves generating a set of candidate geometries at which the flow solver is run to evaluate a hemodynamic metric of interest. The observational dataset thus created can be compactly written as D
 MathType@MTEF@5@5@+=feaafiart1ev1aaatCvAUfKttLearuWrP9MDH5MBPbIqV92AaeXatLxBI9gBaebbnrfifHhDYfgasaacPC6xNi=xH8viVGI8Gi=hEeeu0xXdbba9frFj0xb9qqpG0dXdb9aspeI8k8fiI+fsY=rqGqVepae9pg0db9vqaiVgFr0xfr=xfr=xc9adbaqaaeGacaGaaiaabeqaaeqabiWaaaGcbaWenfgDOvwBHrxAJfwnHbqeg0uy0HwzTfgDPnwy1aaceaGae83aXteaaa@374D@ := {**X**, **y**}, where **X **= {**x**^(1)^, **x**^(2)^, ..., **x**^(*l*)^} ∈ ℝ^*p *× *l *^and **y **= {*y*^(1)^, *y*^(2)^, ..., *y*^(*l*)^} ∈ ℝ^*l*^.

The objective of Gaussian process modeling is to construct a computationally cheap emulator that can be used in lieu of the original computer code. The basic assumption made here is that the observed outputs {*y*^(1)^, *y*^(2)^, ..., *y*^(*l*)^} are realizations of a Gaussian random field with parameterized mean and covariance functions. The assumed model structure used for the emulator can hence be written as

Y(**x**) = *β *+ Z(**x**),

where *β *is an unknown hyperparameter to be estimated from the data and Z(**x**) is a Gaussian stochastic process with zero-mean and covariance

Cov(Z(x),Z(x′))=σz2R(x,x′),
 MathType@MTEF@5@5@+=feaafiart1ev1aaatCvAUfKttLearuWrP9MDH5MBPbIqV92AaeXatLxBI9gBaebbnrfifHhDYfgasaacPC6xNi=xI8qiVKYPFjYdHaVhbbf9v8qqaqFr0xc9vqFj0dXdbba91qpepeI8k8fiI+fsY=rqGqVepae9pg0db9vqaiVgFr0xfr=xfr=xc9adbaqaaeGacaGaaiaabeqaaeqabiWaaaGcbaGaee4qamKaee4Ba8MaeeODayNaeiikaGIaeeOwaOLaeiikaGccbeGae8hEaGNaeiykaKIaeiilaWIaeeOwaOLaeiikaGIaf8hEaGNbauaacqGGPaqkcqGGPaqkcqGH9aqpiiGacqGFdpWCdaqhaaWcbaGaemOEaOhabaGaeGOmaidaaOGaeeOuaiLaeiikaGIae8hEaGNaeiilaWccbaGaf0hEaGNbauaacqGGPaqkcqGGSaalaaa@4881@

where R(**x**, **x**') is a correlation function that can be tuned to the training dataset and σz2
 MathType@MTEF@5@5@+=feaafiart1ev1aaatCvAUfKttLearuWrP9MDH5MBPbIqV92AaeXatLxBI9gBaebbnrfifHhDYfgasaacPC6xNi=xH8viVGI8Gi=hEeeu0xXdbba9frFj0xb9qqpG0dXdb9aspeI8k8fiI+fsY=rqGqVepae9pg0db9vqaiVgFr0xfr=xfr=xc9adbaqaaeGacaGaaiaabeqaaeqabiWaaaGcbaacciGae83Wdm3aa0baaSqaaiabdQha6bqaaiabikdaYaaaaaa@3039@ is the so called process variance which is another parameter to be estimated from the data. Note that by definition, R must be positive-definite. A commonly used choice of correlation function is the stationary family which obeys the *product correlation rule *[44].

R(x,x′)=∏j=1pexp⁡(−θj|xj−x′j|pj),
 MathType@MTEF@5@5@+=feaafiart1ev1aaatCvAUfKttLearuWrP9MDH5MBPbIqV92AaeXatLxBI9gBaebbnrfifHhDYfgasaacPC6xNi=xI8qiVKYPFjYdHaVhbbf9v8qqaqFr0xc9vqFj0dXdbba91qpepeI8k8fiI+fsY=rqGqVepae9pg0db9vqaiVgFr0xfr=xfr=xc9adbaqaaeGacaGaaiaabeqaaeqabiWaaaGcbaGaeeOuaiLaeiikaGccbeGae8hEaGNaeiilaWIaf8hEaGNbauaacqGGPaqkcqGH9aqpdaqeWbqaaiGbcwgaLjabcIha4jabcchaWjabcIcaOiabgkHiTGGaciab+H7aXnaaBaaaleaacqWGQbGAaeqaaOGaeiiFaWNae8hEaG3aaSbaaSqaaiabdQgaQbqabaGccqGHsislcuWF4baEgaqbamaaBaaaleaacqWGQbGAaeqaaOGaeiiFaW3aaWbaaSqabeaacqWGWbaCdaWgaaadbaGaemOAaOgabeaaaaGccqGGPaqkaSqaaiabdQgaQjabg2da9iabigdaXaqaaiabdchaWbqdcqGHpis1aOGaeiilaWcaaa@5310@

where *θ*_*j *_≥ 0, 0 <*p*_*j *_≤ 2, *j *= 1, 2, ..., *p *are the *hyperparameters*. We shall hence use the symbol ***θ ***to denote the vector of hyperparameters. Furthermore, we have chosen *p*_*j *_= 2 to reflect the belief that the underlying function being modeled is smooth and infinitely differentiable. The hyperparameters ***θ ***which control the nonlinearity of the emulator are estimated from the data. For example, small values of *θ*_*j *_indicate that the output is a smooth function of the *j*th variable while large values indicate highly nonlinear behavior. It is also possible to tune the parameters *p*_*j *_to the data which allows for the possibility of modeling functions which may be discontinuous. In theory, the choice of an optimal covariance function is data-dependent. However, in practice it has been found that the parameterized covariance function in Equation (2) offers sufficient flexibility for modeling smooth and highly nonlinear functions [45].

The Bayesian approach to data modeling used here involves two levels of inferencing. The objective of the first level of inferencing is to estimate the unknown hyperparameters from the given observational dataset D
 MathType@MTEF@5@5@+=feaafiart1ev1aaatCvAUfKttLearuWrP9MDH5MBPbIqV92AaeXatLxBI9gBaebbnrfifHhDYfgasaacPC6xNi=xH8viVGI8Gi=hEeeu0xXdbba9frFj0xb9qqpG0dXdb9aspeI8k8fiI+fsY=rqGqVepae9pg0db9vqaiVgFr0xfr=xfr=xc9adbaqaaeGacaGaaiaabeqaaeqabiWaaaGcbaWenfgDOvwBHrxAJfwnHbqeg0uy0HwzTfgDPnwy1aaceaGae83aXteaaa@374D@. This can be done using Bayes theorem which gives

P(θ,β,σz2|D)=P(D|θ,β,σz2)P(θ,β,σz2)P(D),
 MathType@MTEF@5@5@+=feaafiart1ev1aaatCvAUfKttLearuWrP9MDH5MBPbIqV92AaeXatLxBI9gBaebbnrfifHhDYfgasaacPC6xNi=xI8qiVKYPFjYdHaVhbbf9v8qqaqFr0xc9vqFj0dXdbba91qpepeI8k8fiI+fsY=rqGqVepae9pg0db9vqaiVgFr0xfr=xfr=xc9adbaqaaeGacaGaaiaabeqaaeqabiWaaaGcbaGaeeiuaaLaeiikaGcccmGae8hUdeNaeiilaWccciGae4NSdiMaeiilaWIae43Wdm3aa0baaSqaaiabdQha6bqaaiabikdaYaaakiabcYha8nrtHrhAL1wy0L2yHvtyaeHbnfgDOvwBHrxAJfwnaGabaiab9nq8ejabcMcaPiabg2da9KqbaoaalaaabaGaeeiuaaLaeiikaGIae03aXtKaeiiFaWNae8hUdeNaeiilaWIae4NSdiMaeiilaWIae43Wdm3aa0baaeaacqWG6bGEaeaacqaIYaGmaaGaeiykaKIaeeiuaaLaeiikaGIae8hUdeNaeiilaWIae4NSdiMaeiilaWIae43Wdm3aa0baaeaacqWG6bGEaeaacqaIYaGmaaGaeiykaKcabaGaeeiuaaLaeiikaGIae03aXtKaeiykaKcaaOGaeiilaWcaaa@683D@

where P(***θ***, *β*, σz2
 MathType@MTEF@5@5@+=feaafiart1ev1aaatCvAUfKttLearuWrP9MDH5MBPbIqV92AaeXatLxBI9gBaebbnrfifHhDYfgasaacPC6xNi=xH8viVGI8Gi=hEeeu0xXdbba9frFj0xb9qqpG0dXdb9aspeI8k8fiI+fsY=rqGqVepae9pg0db9vqaiVgFr0xfr=xfr=xc9adbaqaaeGacaGaaiaabeqaaeqabiWaaaGcbaacciGae83Wdm3aa0baaSqaaiabdQha6bqaaiabikdaYaaaaaa@3039@|D
 MathType@MTEF@5@5@+=feaafiart1ev1aaatCvAUfKttLearuWrP9MDH5MBPbIqV92AaeXatLxBI9gBaebbnrfifHhDYfgasaacPC6xNi=xH8viVGI8Gi=hEeeu0xXdbba9frFj0xb9qqpG0dXdb9aspeI8k8fiI+fsY=rqGqVepae9pg0db9vqaiVgFr0xfr=xfr=xc9adbaqaaeGacaGaaiaabeqaaeqabiWaaaGcbaWenfgDOvwBHrxAJfwnHbqeg0uy0HwzTfgDPnwy1aaceaGae83aXteaaa@374D@) is the posterior probability of the hyperparameters,

P(D
 MathType@MTEF@5@5@+=feaafiart1ev1aaatCvAUfKttLearuWrP9MDH5MBPbIqV92AaeXatLxBI9gBaebbnrfifHhDYfgasaacPC6xNi=xH8viVGI8Gi=hEeeu0xXdbba9frFj0xb9qqpG0dXdb9aspeI8k8fiI+fsY=rqGqVepae9pg0db9vqaiVgFr0xfr=xfr=xc9adbaqaaeGacaGaaiaabeqaaeqabiWaaaGcbaWenfgDOvwBHrxAJfwnHbqeg0uy0HwzTfgDPnwy1aaceaGae83aXteaaa@374D@|***θ***, *β*, σz2
 MathType@MTEF@5@5@+=feaafiart1ev1aaatCvAUfKttLearuWrP9MDH5MBPbIqV92AaeXatLxBI9gBaebbnrfifHhDYfgasaacPC6xNi=xH8viVGI8Gi=hEeeu0xXdbba9frFj0xb9qqpG0dXdb9aspeI8k8fiI+fsY=rqGqVepae9pg0db9vqaiVgFr0xfr=xfr=xc9adbaqaaeGacaGaaiaabeqaaeqabiWaaaGcbaacciGae83Wdm3aa0baaSqaaiabdQha6bqaaiabikdaYaaaaaa@3039@) is the likelihood, P(***θ***, *β*, σz2
 MathType@MTEF@5@5@+=feaafiart1ev1aaatCvAUfKttLearuWrP9MDH5MBPbIqV92AaeXatLxBI9gBaebbnrfifHhDYfgasaacPC6xNi=xH8viVGI8Gi=hEeeu0xXdbba9frFj0xb9qqpG0dXdb9aspeI8k8fiI+fsY=rqGqVepae9pg0db9vqaiVgFr0xfr=xfr=xc9adbaqaaeGacaGaaiaabeqaaeqabiWaaaGcbaacciGae83Wdm3aa0baaSqaaiabdQha6bqaaiabikdaYaaaaaa@3039@) is the assumed prior for the hyperparameters and P(D
 MathType@MTEF@5@5@+=feaafiart1ev1aaatCvAUfKttLearuWrP9MDH5MBPbIqV92AaeXatLxBI9gBaebbnrfifHhDYfgasaacPC6xNi=xH8viVGI8Gi=hEeeu0xXdbba9frFj0xb9qqpG0dXdb9aspeI8k8fiI+fsY=rqGqVepae9pg0db9vqaiVgFr0xfr=xfr=xc9adbaqaaeGacaGaaiaabeqaaeqabiWaaaGcbaWenfgDOvwBHrxAJfwnHbqeg0uy0HwzTfgDPnwy1aaceaGae83aXteaaa@374D@) is a normalizing constant called the evidence. To ensure computational efficiency, we adopt an empirical Bayesian approach, wherein the posterior distribution of the hyperparameters is approximated by point values obtained by maximizing the likelihood function – see the subsection that follows for details.

Once the hyperparameters have been estimated, the second level of inferencing involves using these values to estimate the value of the unknown function *y*(**x**) at a new point, say **x**. By virtue of the prior that the observed outputs are the realizations of a Gaussian random field, the posterior distribution P(*y*(**x**)|D
 MathType@MTEF@5@5@+=feaafiart1ev1aaatCvAUfKttLearuWrP9MDH5MBPbIqV92AaeXatLxBI9gBaebbnrfifHhDYfgasaacPC6xNi=xH8viVGI8Gi=hEeeu0xXdbba9frFj0xb9qqpG0dXdb9aspeI8k8fiI+fsY=rqGqVepae9pg0db9vqaiVgFr0xfr=xfr=xc9adbaqaaeGacaGaaiaabeqaaeqabiWaaaGcbaWenfgDOvwBHrxAJfwnHbqeg0uy0HwzTfgDPnwy1aaceaGae83aXteaaa@374D@>, *θ*_*j*_, *β*, σz2
 MathType@MTEF@5@5@+=feaafiart1ev1aaatCvAUfKttLearuWrP9MDH5MBPbIqV92AaeXatLxBI9gBaebbnrfifHhDYfgasaacPC6xNi=xH8viVGI8Gi=hEeeu0xXdbba9frFj0xb9qqpG0dXdb9aspeI8k8fiI+fsY=rqGqVepae9pg0db9vqaiVgFr0xfr=xfr=xc9adbaqaaeGacaGaaiaabeqaaeqabiWaaaGcbaacciGae83Wdm3aa0baaSqaaiabdQha6bqaaiabikdaYaaaaaa@3039@) is Gaussian [46], i.e., the prediction of the emulator at a point **x **can be written as Y(**x**) ~ N
 MathType@MTEF@5@5@+=feaafiart1ev1aaatCvAUfKttLearuWrP9MDH5MBPbIqV92AaeXatLxBI9gBaebbnrfifHhDYfgasaacPC6xNi=xH8viVGI8Gi=hEeeu0xXdbba9frFj0xb9qqpG0dXdb9aspeI8k8fiI+fsY=rqGqVepae9pg0db9vqaiVgFr0xfr=xfr=xc9adbaqaaeGacaGaaiaabeqaaeqabiWaaaGcbaWenfgDOvwBHrxAJfwnHbqeg0uy0HwzTfgDPnwy1aaceaGae8xdX7eaaa@3761@(Y^
 MathType@MTEF@5@5@+=feaafiart1ev1aaatCvAUfKttLearuWrP9MDH5MBPbIqV92AaeXatLxBI9gBaebbnrfifHhDYfgasaacPC6xNi=xH8viVGI8Gi=hEeeu0xXdbba9frFj0xb9qqpG0dXdb9aspeI8k8fiI+fsY=rqGqVepae9pg0db9vqaiVgFr0xfr=xfr=xc9adbaqaaeGacaGaaiaabeqaaeqabiWaaaGcbaacbaGaf8xwaKLbaKaaaaa@2D23@(**x**), C(**x**, **x**')). After some algebraic manipulations (see, for example, [27]), the posterior mean and covariance can be written as follows:

Y^(x)=β+r(x)TR−1(y−1β^),
 MathType@MTEF@5@5@+=feaafiart1ev1aaatCvAUfKttLearuWrP9MDH5MBPbIqV92AaeXatLxBI9gBaebbnrfifHhDYfgasaacPC6xNi=xI8qiVKYPFjYdHaVhbbf9v8qqaqFr0xc9vqFj0dXdbba91qpepeI8k8fiI+fsY=rqGqVepae9pg0db9vqaiVgFr0xfr=xfr=xc9adbaqaaeGacaGaaiaabeqaaeqabiWaaaGcbaGafeywaKLbaKaacqGGOaakieqacqWF4baEcqGGPaqkcqGH9aqpiiGacqGFYoGycqGHRaWkcqWFYbGCcqGGOaakcqWF4baEcqGGPaqkdaahaaWcbeqaaiabdsfaubaakiab=jfasnaaCaaaleqabaGaeyOeI0IaeGymaedaaOWaaeWaaeaacqWF5bqEcqGHsislcqWFXaqmcuGFYoGygaqcaaGaayjkaiaawMcaaiabcYcaSaaa@44BE@

and

C(x,x′)=σz2(R(x,x′)−r(x)TR−1r(x′)),
 MathType@MTEF@5@5@+=feaafiart1ev1aaatCvAUfKttLearuWrP9MDH5MBPbIqV92AaeXatLxBI9gBaebbnrfifHhDYfgasaacPC6xNi=xI8qiVKYPFjYdHaVhbbf9v8qqaqFr0xc9vqFj0dXdbba91qpepeI8k8fiI+fsY=rqGqVepae9pg0db9vqaiVgFr0xfr=xfr=xc9adbaqaaeGacaGaaiaabeqaaeqabiWaaaGcbaGaee4qamKaeiikaGccbeGae8hEaGNaeiilaWIaf8hEaGNbauaacqGGPaqkcqGH9aqpiiGacqGFdpWCdaqhaaWcbaGaemOEaOhabaGaeGOmaidaaOGaeiikaGIaeeOuaiLaeiikaGIae8hEaGNaeiilaWIaf8hEaGNbauaacqGGPaqkcqGHsislcqWFYbGCcqGGOaakcqWF4baEcqGGPaqkdaahaaWcbeqaaiabdsfaubaakiab=jfasnaaCaaaleqabaGaeyOeI0IaeGymaedaaOGae8NCaiNaeiikaGIaf8hEaGNbauaacqGGPaqkcqGGPaqkcqGGSaalaaa@5038@

where **R **∈ ℝ^*l *× *l *^is the correlation matrix computed using the training points whose *ij*th element is computed as ***R***_*ij *_= R(**x**^(*i*)^, **x**^(*j*)^). **r**(**x**) = {R(**x**, **x**^(1)^), R(**x**, **x**^(2)^), ..., R(**x**, **x**^(*l*)^)} ∈ ℝ^*l *^is the correlation between the new point **x **and the training points, and **1 **= {1, 1, ..., 1} ∈ ℝ^*l*^. It can be observed from Equations (5) and (6) that the Bayesian inferencing approach ultimately leads to an approximation of the computer code as a multidimensional Gaussian random field. The posterior variance computed using Equation (6), i.e., *C*(**x**, **x**), can be interpreted as an estimate of the uncertainty involved in making predictions at a new point **x**. Note that this uncertainty arises from the fact that only a finite set of points are used to construct the emulator. The quantification of uncertainty in the output due to variability in the inputs will be dealt with in the next section.

In practice, for the sake of computational efficiency, we compute the Cholesky decomposition of **R**. This allows the posterior mean to be computed at any point of interest using a vector-vector product, i.e., Y^
 MathType@MTEF@5@5@+=feaafiart1ev1aaatCvAUfKttLearuWrP9MDH5MBPbIqV92AaeXatLxBI9gBaebbnrfifHhDYfgasaacPC6xNi=xH8viVGI8Gi=hEeeu0xXdbba9frFj0xb9qqpG0dXdb9aspeI8k8fiI+fsY=rqGqVepae9pg0db9vqaiVgFr0xfr=xfr=xc9adbaqaaeGacaGaaiaabeqaaeqabiWaaaGcbaacbaGaf8xwaKLbaKaaaaa@2D23@(**x**) = *β *+ **r**(**x**)^*T*^**w**, where **w **= **R**^-1^(**y **- **1**β^
 MathType@MTEF@5@5@+=feaafiart1ev1aaatCvAUfKttLearuWrP9MDH5MBPbIqV92AaeXatLxBI9gBaebbnrfifHhDYfgasaacPC6xNi=xH8viVGI8Gi=hEeeu0xXdbba9frFj0xb9qqpG0dXdb9aspeI8k8fiI+fsY=rqGqVepae9pg0db9vqaiVgFr0xfr=xfr=xc9adbaqaaeGacaGaaiaabeqaaeqabiWaaaGcbaacciGaf8NSdiMbaKaaaaa@2D8B@). However, the computation of the variance (or error bar) of the posterior process (i.e., C(**x**, **x**)) requires a forward and back substitution.

#### Maximum likelihood estimation

As described previously, Equation 4 can be used to estimate the posterior distribution of the hyperparameters, for example, using Markov chain Monte Carlo simulation techniques [25,26]. In practice, however, an empirical Bayesian approach is computationally more efficient. In this approach, the hyperparameters ***θ ***= {*θ*_*j*_}, *j *= 1, 2, ..., *p*, *β*, and σz2
 MathType@MTEF@5@5@+=feaafiart1ev1aaatCvAUfKttLearuWrP9MDH5MBPbIqV92AaeXatLxBI9gBaebbnrfifHhDYfgasaacPC6xNi=xH8viVGI8Gi=hEeeu0xXdbba9frFj0xb9qqpG0dXdb9aspeI8k8fiI+fsY=rqGqVepae9pg0db9vqaiVgFr0xfr=xfr=xc9adbaqaaeGacaGaaiaabeqaaeqabiWaaaGcbaacciGae83Wdm3aa0baaSqaaiabdQha6bqaaiabikdaYaaaaaa@3039@ which arise in the correlation function defined in Equation (2) are estimated by maximizing the likelihood function. Maximization of the likelihood function leads to those values of the hyperparameters that are most likely to have generated the training dataset. Since we invoked the prior that the observed outputs are the realizations of a Gaussian random field, the likelihood function is given by

P(D|θ,β,σz2)=exp⁡(−(y−1β)TR−1(y−1β)2σz2)(2πσz2)l/2|R|1/2.
 MathType@MTEF@5@5@+=feaafiart1ev1aaatCvAUfKttLearuWrP9MDH5MBPbIqV92AaeXatLxBI9gBaebbnrfifHhDYfgasaacPC6xNi=xI8qiVKYPFjYdHaVhbbf9v8qqaqFr0xc9vqFj0dXdbba91qpepeI8k8fiI+fsY=rqGqVepae9pg0db9vqaiVgFr0xfr=xfr=xc9adbaqaaeGacaGaaiaabeqaaeqabiWaaaGcbaGaeeiuaaLaeiikaGYenfgDOvwBHrxAJfwnHbqeg0uy0HwzTfgDPnwy1aaceaGae83aXtKaeiiFaWhccmGae4hUdeNaeiilaWccciGae0NSdiMaeiilaWIae03Wdm3aa0baaSqaaiabdQha6bqaaiabikdaYaaakiabcMcaPiabg2da9KqbaoaalaaabaGagiyzauMaeiiEaGNaeiiCaa3aaeWaaeaacqGHsisldaWcaaqaaiabcIcaOGqabiab8Lha5jabgkHiTiab8fdaXiab9j7aIjabcMcaPmaaCaaabeqaaiabdsfaubaacqaFsbGudaahaaqabeaacqGHsislcqaIXaqmaaGaeiikaGIaeWxEaKNaeyOeI0IaeWxmaeJae0NSdiMaeiykaKcabaGaeGOmaiJae03Wdm3aa0baaeaacqWG6bGEaeaacqaIYaGmaaaaaaGaayjkaiaawMcaaaqaamaabmaabaGaeGOmaiJae0hWdaNae03Wdm3aa0baaeaacqWG6bGEaeaacqaIYaGmaaaacaGLOaGaayzkaaWaaWbaaeqabaGaemiBaWMaei4la8IaeGOmaidaamaaemaabaGaeWNuaifacaGLhWUaayjcSdWaaWbaaeqabaGaeGymaeJaei4la8IaeGOmaidaaaaacqGGUaGlaaa@78A9@

The negative log-likelihood function to be minimized (after dropping out constant terms that do not depend on the hyperparameters) can be written as

L(θ,β,σz2)=−[lln⁡σz2+ln⁡|R|+(y−1β)TR−1(y−1β)σz2]2.
 MathType@MTEF@5@5@+=feaafiart1ev1aaatCvAUfKttLearuWrP9MDH5MBPbIqV92AaeXatLxBI9gBaebbnrfifHhDYfgasaacPC6xNi=xI8qiVKYPFjYdHaVhbbf9v8qqaqFr0xc9vqFj0dXdbba91qpepeI8k8fiI+fsY=rqGqVepae9pg0db9vqaiVgFr0xfr=xfr=xc9adbaqaaeGacaGaaiaabeqaaeqabiWaaaGcbaGaemitaWKaeiikaGcccmGae8hUdeNaeiilaWccciGae4NSdiMaeiilaWIae43Wdm3aa0baaSqaaiabdQha6bqaaiabikdaYaaakiabcMcaPiabg2da9iabgkHiTKqbaoaalaaabaWaamWaaeaacqWGSbaBcyGGSbaBcqGGUbGBcqGFdpWCdaqhaaqaaiabdQha6bqaaiabikdaYaaacqGHRaWkcyGGSbaBcqGGUbGBdaabdaqaaGqabiab9jfasbGaay5bSlaawIa7aiabgUcaRmaalaaabaGaeiikaGIae0xEaKNaeyOeI0Iae0xmaeJae4NSdiMaeiykaKYaaWbaaeqabaGaemivaqfaaiab9jfasnaaCaaabeqaaiabgkHiTiabigdaXaaacqGGOaakcqqF5bqEcqGHsislcqqFXaqmcqGFYoGycqGGPaqkaeaacqGFdpWCdaqhaaqaaiabdQha6bqaaiabikdaYaaaaaaacaGLBbGaayzxaaaabaGaeGOmaidaaOGaeiOla4caaa@6630@

Numerical optimization techniques are required for the maximization of Equation (8) to estimate the unknown parameters; see reference [27] for a detailed discussion of the computational and implementation aspects. Since computing *L*(***θ***, *β*, σz2
 MathType@MTEF@5@5@+=feaafiart1ev1aaatCvAUfKttLearuWrP9MDH5MBPbIqV92AaeXatLxBI9gBaebbnrfifHhDYfgasaacPC6xNi=xH8viVGI8Gi=hEeeu0xXdbba9frFj0xb9qqpG0dXdb9aspeI8k8fiI+fsY=rqGqVepae9pg0db9vqaiVgFr0xfr=xfr=xc9adbaqaaeGacaGaaiaabeqaaeqabiWaaaGcbaacciGae83Wdm3aa0baaSqaaiabdQha6bqaaiabikdaYaaaaaa@3039@) and its gradients involves computing and decomposing the dense *l *× *l *covariance matrix **R **(requiring O
 MathType@MTEF@5@5@+=feaafiart1ev1aaatCvAUfKttLearuWrP9MDH5MBPbIqV92AaeXatLxBI9gBaebbnrfifHhDYfgasaacPC6xNi=xH8viVGI8Gi=hEeeu0xXdbba9frFj0xb9qqpG0dXdb9aspeI8k8fiI+fsY=rqGqVepae9pg0db9vqaiVgFr0xfr=xfr=xc9adbaqaaeGacaGaaiaabeqaaeqabiWaaaGcbaWenfgDOvwBHrxAJfwnHbqeg0uy0HwzTfgDPnwy1aaceaGae8NdX=eaaa@3763@(*l*^3^) resources) at each iteration, maximum likelihood estimation can be prohibitively expensive even for moderately sized data, e.g., say a few thousand points. Recent work to address this issue has led to data parallel techniques [47] which can cope with large datasets. In the context of the present study, due to the high computational cost involved in solving the Navier-Stokes equations, only a modestly sized training dataset is available for model building. As a consequence, the computational cost associated with maximization of *L*(***θ***, *β*, σz2
 MathType@MTEF@5@5@+=feaafiart1ev1aaatCvAUfKttLearuWrP9MDH5MBPbIqV92AaeXatLxBI9gBaebbnrfifHhDYfgasaacPC6xNi=xH8viVGI8Gi=hEeeu0xXdbba9frFj0xb9qqpG0dXdb9aspeI8k8fiI+fsY=rqGqVepae9pg0db9vqaiVgFr0xfr=xfr=xc9adbaqaaeGacaGaaiaabeqaaeqabiWaaaGcbaacciGae83Wdm3aa0baaSqaaiabdQha6bqaaiabikdaYaaaaaa@3039@) is negligible.

It is worth noting here that for some datasets the correlation matrix **R **may be ill-conditioned, particularly when two or more training points lie close to each other. To circumvent this difficulty, we add a small term (say 10^-6^) to the diagonal elements of **R**. A more robust strategy would be to employ a singular value decomposition of **R **– this would however be computationally expensive for large datasets.

#### Model validation

After emulator construction, validation studies will help in assessing how well the approximate model agrees with the true model. A brute force approach would be to run the numerical simulation for a number of additional geometries (testing points) and check how well the approximate model correlates at these points. In most practical applications, generation of additional testing data is computationally expensive. This motivates the development of alternative model diagnostic measures that can be evaluated more cheaply.

The accuracy of the prediction error estimate (i.e., the posterior variance *C*(**x**, **x**)) depends on the validity of the assumption made in creating the emulator; namely that a Gaussian process prior is appropriate for the simulator under consideration. Techniques for model diagnostics available in the literature [48] can be applied to check the validity of this assumption and the accuracy of the approximation. One such measure is the *standardized cross validated residual *(SCVR) defined below

SCVRi=y(x(i))−Y^−i(x(i))C−i(x(i),x(i)),i=1,2,...,l,
 MathType@MTEF@5@5@+=feaafiart1ev1aaatCvAUfKttLearuWrP9MDH5MBPbIqV92AaeXatLxBI9gBaebbnrfifHhDYfgasaacPC6xNi=xI8qiVKYPFjYdHaVhbbf9v8qqaqFr0xc9vqFj0dXdbba91qpepeI8k8fiI+fsY=rqGqVepae9pg0db9vqaiVgFr0xfr=xfr=xc9adbaqaaeGacaGaaiaabeqaaeqabiWaaaGcbaqbaeqabeGaaaqaaiabbofatjabboeadjabbAfawjabbkfasnaaBaaaleaacqWGPbqAaeqaaOGaeyypa0tcfa4aaSaaaeaacqWG5bqEcqGGOaakieqacqWF4baEdaahaaqabeaacqGGOaakcqWGPbqAcqGGPaqkaaGaeiykaKIaeyOeI0IafeywaKLbaKaadaWgaaqaaiabgkHiTiabdMgaPbqabaGaeiikaGIae8hEaG3aaWbaaeqabaGaeiikaGIaemyAaKMaeiykaKcaaiabcMcaPaqaamaakaaabaGaem4qam0aaSbaaeaacqGHsislcqWGPbqAaeqaaiabcIcaOiab=Hha4naaCaaabeqaaiabcIcaOiabdMgaPjabcMcaPaaacqGGSaalcqWF4baEdaahaaqabeaacqGGOaakcqWGPbqAcqGGPaqkaaGaeiykaKcabeaaaaGccqGGSaalaeaacqWGPbqAcqGH9aqpcqaIXaqmcqGGSaalcqaIYaGmcqGGSaalcqGGUaGlcqGGUaGlcqGGUaGlcqGGSaalcqWGSbaBcqGGSaalaaaaaa@62E7@

where Y^
 MathType@MTEF@5@5@+=feaafiart1ev1aaatCvAUfKttLearuWrP9MDH5MBPbIqV92AaeXatLxBI9gBaebbnrfifHhDYfgasaacPC6xNi=xH8viVGI8Gi=hEeeu0xXdbba9frFj0xb9qqpG0dXdb9aspeI8k8fiI+fsY=rqGqVepae9pg0db9vqaiVgFr0xfr=xfr=xc9adbaqaaeGacaGaaiaabeqaaeqabiWaaaGcbaacbaGaf8xwaKLbaKaaaaa@2D23@_-*i*_(**x**) and *C*_-*i*_(**x**, **x**) denotes the mean and variance of the prediction at a point **x **without using the *i*^*th *^training point. SCVR_*i *_can be computed for all the training points by removing the contribution of the corresponding point from the correlation matrix **R**.

In the cross validation procedure, it is generally assumed that the maximum likelihood estimates for the hyperparameters do not change when one training point is removed from the training set. If the Gaussian process prior is appropriate for the problem under consideration, SCVR_*i *_will roughly lie in the interval [-3, +3]. This implies that given the posterior predictions of the emulator at a new design point **x**, the actual output value lies in the interval [Y^(x)−3C(x,x),Y^(x)+3C(x,x)]
 MathType@MTEF@5@5@+=feaafiart1ev1aaatCvAUfKttLearuWrP9MDH5MBPbIqV92AaeXatLxBI9gBaebbnrfifHhDYfgasaacPC6xNi=xH8viVGI8Gi=hEeeu0xXdbba9frFj0xb9qqpG0dXdb9aspeI8k8fiI+fsY=rqGqVepae9pg0db9vqaiVgFr0xfr=xfr=xc9adbaqaaeGacaGaaiaabeqaaeqabiWaaaGcbaGaei4waSLafeywaKLbaKaacqGGOaakieqacqWF4baEcqGGPaqkcqGHsislcqaIZaWmdaGcaaqaaiabdoeadjabcIcaOiab=Hha4jabcYcaSiab=Hha4jabcMcaPaWcbeaakiabcYcaSiqbbMfazzaajaGaeiikaGIae8hEaGNaeiykaKIaey4kaSIaeG4mamZaaOaaaeaacqWGdbWqcqGGOaakcqWF4baEcqGGSaalcqWF4baEcqGGPaqkaSqabaGccqGGDbqxaaa@4934@ with a high level of confidence. More details and additional validation procedures such as leave-one-out and leave-two-out validation can be found in [27].

### Post processing of the emulator

Once the Bayesian Gaussian process emulator has been constructed and validated, it can be mined in the post-processing phase. In this section, we describe how the emulator can be applied to: (i) compute statistics of the output when the inputs are modeled as random variables with a specified joint probability distribution, (ii) identify the relative importance of each input variable and (iii) identify which settings of the inputs lead to maximum and minimum values of the output.

#### Uncertainty analysis

The Monte Carlo simulation (MCS) technique is commonly used to approximate the statistical moments of implicit functions of the form *y*(**x**) when the elements of **x **are random variables following a given distribution. To illustrate the MCS technique, consider the following multi-dimensional integral given by

I=〈y(x)〉=∫χy(x)P(x)dx,
 MathType@MTEF@5@5@+=feaafiart1ev1aaatCvAUfKttLearuWrP9MDH5MBPbIqV92AaeXatLxBI9gBaebbnrfifHhDYfgasaacPC6xNi=xI8qiVKYPFjYdHaVhbbf9v8qqaqFr0xc9vqFj0dXdbba91qpepeI8k8fiI+fsY=rqGqVepae9pg0db9vqaiVgFr0xfr=xfr=xc9adbaqaaeGacaGaaiaabeqaaeqabiWaaaGcbaGaemysaKKaeyypa0JaeyykJeUaemyEaKNaeiikaGccbeGae8hEaGNaeiykaKIaeyOkJeVaeyypa0Zaa8qeaeaacqWG5bqEcqGGOaakcqWF4baEcqGGPaqkcqqGqbaucqGGOaakcqWF4baEcqGGPaqkcqqGKbazcqWF4baEaSqaaGGaciab+D8aJbqab0Gaey4kIipakiabcYcaSaaa@47DC@

where *y*(**x**) is a function calculated by running an expensive computer model.

In the MCS technique, *y*(**x**) is evaluated at numerous points by drawing samples from the distribution P(**x**) and the integral is subsequently approximated as I≈(1/m)∑i=1my(x(i))
 MathType@MTEF@5@5@+=feaafiart1ev1aaatCvAUfKttLearuWrP9MDH5MBPbIqV92AaeXatLxBI9gBaebbnrfifHhDYfgasaacPC6xNi=xH8viVGI8Gi=hEeeu0xXdbba9frFj0xb9qqpG0dXdb9aspeI8k8fiI+fsY=rqGqVepae9pg0db9vqaiVgFr0xfr=xfr=xc9adbaqaaeGacaGaaiaabeqaaeqabiWaaaGcbaGaemysaKKaeyisISRaeiikaGIaeGymaeJaei4la8IaemyBa0MaeiykaKYaaabmaeaacqWG5bqEcqGGOaakieqacqWF4baEdaahaaWcbeqaaiabcIcaOiabdMgaPjabcMcaPaaakiabcMcaPaWcbaGaemyAaKMaeyypa0JaeGymaedabaGaemyBa0ganiabggHiLdaaaa@4230@. The convergence rate of the Monte Carlo estimate is O(1m)
 MathType@MTEF@5@5@+=feaafiart1ev1aaatCvAUfKttLearuWrP9MDH5MBPbIqV92AaeXatLxBI9gBaebbnrfifHhDYfgasaacPC6xNi=xH8viVGI8Gi=hEeeu0xXdbba9frFj0xb9qqpG0dXdb9aspeI8k8fiI+fsY=rqGqVepae9pg0db9vqaiVgFr0xfr=xfr=xc9adbaqaaeGacaGaaiaabeqaaeqabiWaaaGcbaWenfgDOvwBHrxAJfwnHbqeg0uy0HwzTfgDPnwy1aaceaGae8NdX=0aaeWaaKqbagaadaWcaaqaaiabigdaXaqaamaakaaabaGaemyBa0gabeaaaaaakiaawIcacaGLPaaaaaa@3BF7@. Hence, to ensure accurate approximations, a large sample size becomes necessary; for example, to improve accuracy by one decimal place, around 100 times more samples will be required. Another drawback of the MCS technique is that it wastes information since the realizations of the inputs **x**^(*i*)^, are not used in the final estimate [49]. It is worth noting here that it is possible to employ quasi-MCS techniques that enjoy faster convergence rates [27]. However, even so, the resulting reductions in computational cost are often not very significant. The emulator-assisted uncertainty analysis approach discussed below presents a computationally more efficient alternative to simulation techniques. Further, the emulator based approach uses both the input realizations **x**^(*i*) ^and the corresponding output values to estimate ⟨*y*(**x**)⟩.

Consider the case when the inputs of the simulation code are modeled as random variables with joint probability density function P(**x**) and it is sought to approximate the first two statistical moments of the computer code output *y*(**x**). We discuss next how a Gaussian process emulator can be used to approximate the first two statistical moments of *y*(**x**). Recall from Equations (9) and (10) that the emulator is itself a Gaussian random field, i.e., *Y*(**x**) ~ N
 MathType@MTEF@5@5@+=feaafiart1ev1aaatCvAUfKttLearuWrP9MDH5MBPbIqV92AaeXatLxBI9gBaebbnrfifHhDYfgasaacPC6xNi=xH8viVGI8Gi=hEeeu0xXdbba9frFj0xb9qqpG0dXdb9aspeI8k8fiI+fsY=rqGqVepae9pg0db9vqaiVgFr0xfr=xfr=xc9adbaqaaeGacaGaaiaabeqaaeqabiWaaaGcbaWenfgDOvwBHrxAJfwnHbqeg0uy0HwzTfgDPnwy1aaceaGae8xdX7eaaa@3761@(Y^
 MathType@MTEF@5@5@+=feaafiart1ev1aaatCvAUfKttLearuWrP9MDH5MBPbIqV92AaeXatLxBI9gBaebbnrfifHhDYfgasaacPC6xNi=xH8viVGI8Gi=hEeeu0xXdbba9frFj0xb9qqpG0dXdb9aspeI8k8fiI+fsY=rqGqVepae9pg0db9vqaiVgFr0xfr=xfr=xc9adbaqaaeGacaGaaiaabeqaaeqabiWaaaGcbaacbaGaf8xwaKLbaKaaaaa@2D23@(**x**), *C*(**x**, **x**)). As a consequence the statistical moments of the emulator have to be described using random variables [25,27]. More explicitly, the mean of the emulator output is a Gaussian random variable whose statistics can be computed as follows

K=∫χY(x)P(x)dx~N(〈K〉,σK2),
 MathType@MTEF@5@5@+=feaafiart1ev1aaatCvAUfKttLearuWrP9MDH5MBPbIqV92AaeXatLxBI9gBaebbnrfifHhDYfgasaacPC6xNi=xI8qiVKYPFjYdHaVhbbf9v8qqaqFr0xc9vqFj0dXdbba91qpepeI8k8fiI+fsY=rqGqVepae9pg0db9vqaiVgFr0xfr=xfr=xc9adbaqaaeGacaGaaiaabeqaaeqabiWaaaGcbaGaee4saSKaeyypa0Zaa8qeaeaacqqGzbqwcqGGOaakieqacqWF4baEcqGGPaqkcqqGqbaucqGGOaakcqWF4baEcqGGPaqkcqqGKbazcqWF4baEaSqaaGGaciab+D8aJbqab0Gaey4kIipakiabc6ha+nrtHrhAL1wy0L2yHvtyaeHbnfgDOvwBHrxAJfwnaGabaiab91q8ojabcIcaOiabgMYiHlabbUealjabgQYiXlabcYcaSiab+n8aZnaaDaaaleaacqqGlbWsaeaacqaIYaGmaaGccqGGPaqkcqGGSaalaaa@56B6@

where

〈K〉=∫χY^(x)P(x)dx,
 MathType@MTEF@5@5@+=feaafiart1ev1aaatCvAUfKttLearuWrP9MDH5MBPbIqV92AaeXatLxBI9gBaebbnrfifHhDYfgasaacPC6xNi=xI8qiVKYPFjYdHaVhbbf9v8qqaqFr0xc9vqFj0dXdbba91qpepeI8k8fiI+fsY=rqGqVepae9pg0db9vqaiVgFr0xfr=xfr=xc9adbaqaaeGacaGaaiaabeqaaeqabiWaaaGcbaGaeyykJeUaee4saSKaeyOkJeVaeyypa0Zaa8qeaeaacuqGzbqwgaqcaiabcIcaOGqabiab=Hha4jabcMcaPiabbcfaqjabcIcaOiab=Hha4jabcMcaPiabbsgaKjab=Hha4bWcbaacciGae43XdmgabeqdcqGHRiI8aOGaeiilaWcaaa@4204@

σK2=∫χ∫χC(x,x′)P(x)P(x′)dxdx′,
 MathType@MTEF@5@5@+=feaafiart1ev1aaatCvAUfKttLearuWrP9MDH5MBPbIqV92AaeXatLxBI9gBaebbnrfifHhDYfgasaacPC6xNi=xI8qiVKYPFjYdHaVhbbf9v8qqaqFr0xc9vqFj0dXdbba91qpepeI8k8fiI+fsY=rqGqVepae9pg0db9vqaiVgFr0xfr=xfr=xc9adbaqaaeGacaGaaiaabeqaaeqabiWaaaGcbaacciGae83Wdm3aa0baaSqaaiabbUealbqaaiabikdaYaaakiabg2da9maapebabaWaa8qeaeaacqqGdbWqcqGGOaakieqacqGF4baEcqGGSaalcuGF4baEgaqbaiabcMcaPiabbcfaqjabcIcaOiab+Hha4jabcMcaPiabbcfaqjabcIcaOiqb+Hha4zaafaGaeiykaKIaeeizaqMae4hEaGNaeeizaqMaf4hEaGNbauaaaSqaaiab=D8aJbqab0Gaey4kIipaaSqaaiab=D8aJbqab0Gaey4kIipakiabcYcaSaaa@4E6E@

and *χ *denotes the support of the distribution function P(**x**).

Similarly, the posterior variance of the emulator is again a random variable, whose mean and variance can be computed as

〈L〉=〈∫χY2(x)P(x)dx〉−σK2−〈K〉2,
 MathType@MTEF@5@5@+=feaafiart1ev1aaatCvAUfKttLearuWrP9MDH5MBPbIqV92AaeXatLxBI9gBaebbnrfifHhDYfgasaacPC6xNi=xI8qiVKYPFjYdHaVhbbf9v8qqaqFr0xc9vqFj0dXdbba91qpepeI8k8fiI+fsY=rqGqVepae9pg0db9vqaiVgFr0xfr=xfr=xc9adbaqaaeGacaGaaiaabeqaaeqabiWaaaGcbaGaeyykJeUaeeitaWKaeyOkJeVaeyypa0ZaaaWabeaadaWdraqaaiabbMfaznaaCaaaleqabaGaeGOmaidaaOGaeiikaGccbeGae8hEaGNaeiykaKIaeeiuaaLaeiikaGIae8hEaGNaeiykaKIaeeizaqMae8hEaGhaleaaiiGacqGFhpWyaeqaniabgUIiYdaakiaawMYicaGLQmcacqGHsislcqGFdpWCdaqhaaWcbaGaee4saSeabaGaeGOmaidaaOGaeyOeI0IaeyykJeUaee4saSKaeyOkJe=aaWbaaSqabeaacqaIYaGmaaGccqGGSaalaaa@5096@

and

σL2=〈∫χY2(x)P(x)dx∫χY2(z)P(z)dz〉−2〈K2∫χY2(x)P(x)dx〉−〈K4〉−〈L〉2.
 MathType@MTEF@5@5@+=feaafiart1ev1aaatCvAUfKttLearuWrP9MDH5MBPbIqV92AaeXatLxBI9gBaebbnrfifHhDYfgasaacPC6xNi=xI8qiVKYPFjYdHaVhbbf9v8qqaqFr0xc9vqFj0dXdbba91qpepeI8k8fiI+fsY=rqGqVepae9pg0db9vqaiVgFr0xfr=xfr=xc9adbaqaaeGacaGaaiaabeqaaeqabiWaaaGcbaqbaeaabiqaaaqaaGGaciab=n8aZnaaDaaaleaacqqGmbataeaacqaIYaGmaaGccqGH9aqpdaaadeqaamaapebabaGaeeywaK1aaWbaaSqabeaacqaIYaGmaaGccqGGOaakieqacqGF4baEcqGGPaqkcqqGqbaucqGGOaakcqGF4baEcqGGPaqkcqqGKbazcqGF4baEaSqaaiab=D8aJbqab0Gaey4kIipakmaapebabaGaeeywaK1aaWbaaSqabeaacqaIYaGmaaGccqGGOaakcqGF6bGEcqGGPaqkcqqGqbaucqGGOaakcqGF6bGEcqGGPaqkcqqGKbazcqGF6bGEaSqaaiab=D8aJbqab0Gaey4kIipaaOGaayzkJiaawQYiaaqaaiabgkHiTiabikdaYmaaamqabaGaee4saS0aaWbaaSqabeaacqaIYaGmaaGcdaWdraqaaiabbMfaznaaCaaaleqabaGaeGOmaidaaOGaeiikaGIae4hEaGNaeiykaKIaeeiuaaLaeiikaGIae4hEaGNaeiykaKIaeeizaqMae4hEaGhaleaacqWFhpWyaeqaniabgUIiYdaakiaawMYicaGLQmcacqGHsislcqGHPms4cqqGlbWsdaahaaWcbeqaaiabisda0aaakiabgQYiXlabgkHiTiabgMYiHlabbYeamjabgQYiXpaaCaaaleqabaGaeGOmaidaaOGaeiOla4caaaaa@7892@

It is to be noted here that the multidimensional integrals in the preceding equations can be analytically computed if the elements of the input vector **x **are uncorrelated Gaussian random variables [50]. For more general distributions, simulation techniques can be applied to compute various statistics of interest including the probability distribution functions. This can be done efficiently since the prediction of the output at a new point **x **using the emulator is computationally very cheap. Furthermore, it is also possible to exploit the emulator sensitivities which are readily computable in order to accelerate the convergence of simulation schemes [51].

#### Sensitivity analysis

It can be seen from Equations (5) and (6) that the Bayesian model does not explicitly reveal the input-output relationships in a readily interpretable way. Consequently, this predictor is not suitable for explaining the functional relationship between the covariates and the response. In order to identify this relationship, the effect of each input needs to be isolated from the others. The response can be decomposed into main effects for each input and this main effect of the *i*th input variable which can be obtained by integrating out the other factors is defined as follows:

γi(xi)=1V∫Y(x)∏h≠idxh.
 MathType@MTEF@5@5@+=feaafiart1ev1aaatCvAUfKttLearuWrP9MDH5MBPbIqV92AaeXatLxBI9gBaebbnrfifHhDYfgasaacPC6xNi=xI8qiVKYPFjYdHaVhbbf9v8qqaqFr0xc9vqFj0dXdbba91qpepeI8k8fiI+fsY=rqGqVepae9pg0db9vqaiVgFr0xfr=xfr=xc9adbaqaaeGacaGaaiaabeqaaeqabiWaaaGcbaacciGae83SdC2aaSbaaSqaaiabdMgaPbqabaGccqGGOaakcqWG4baEdaWgaaWcbaGaemyAaKgabeaakiabcMcaPiabg2da9KqbaoaalaaabaGaeGymaedabaGaeeOvayfaaOWaa8qaaeaacqqGzbqwcqGGOaakieqacqGF4baEcqGGPaqkdaqeqbqaaiabbsgaKjabdIha4naaBaaaleaacqWGObaAaeqaaaqaaiabdIgaOjabgcMi5kabdMgaPbqab0Gaey4dIunaaSqabeqaniabgUIiYdGccqGGUaGlaaa@4A0E@

The above integral can be numerically computed by approximating it by a sum over a grid of *m *points *x*_*h*_^(1)^, ..., *x*_*h*_^(*m*)^. These points can be generated using the Latin hypercube sampling (LHS) technique [52]. Note that since the Bayesian emulator also provides the posterior variance, error bars on the main effects can be readily computed. Similarly, the effect of two or more covariates (joint effects) can be investigated by integrating out all the other covariates or by fixing the other covariates at some values [44,53].

To allow effective presentation of the sensitivity trends, particularly for problems with many variables, it is often useful to condense the main effects into scalar sensitivity factors. One such proposal originally suggested by Sobol [54] involves calculating the following sensitivity metric for each variable in terms of the main effect *γ*_*i *_defined earlier.

Si=∫γi2(xi)dxi∑j=1p∫γj2(xj)dxj.
 MathType@MTEF@5@5@+=feaafiart1ev1aaatCvAUfKttLearuWrP9MDH5MBPbIqV92AaeXatLxBI9gBaebbnrfifHhDYfgasaacPC6xNi=xI8qiVKYPFjYdHaVhbbf9v8qqaqFr0xc9vqFj0dXdbba91qpepeI8k8fiI+fsY=rqGqVepae9pg0db9vqaiVgFr0xfr=xfr=xc9adbaqaaeGacaGaaiaabeqaaeqabiWaaaGcbaGaem4uam1aaSbaaSqaaiabdMgaPbqabaGccqGH9aqpjuaGdaWcaaqaamaapeaabaacciGae83SdC2aa0baaeaacqWGPbqAaeaacqaIYaGmaaGaeiikaGIaemiEaG3aaSbaaeaacqWGPbqAaeqaaiabcMcaPiabbsgaKjabdIha4naaBaaabaGaemyAaKgabeaaaeqabeGaey4kIipaaeaadaaeWaqaamaapeaabaGae83SdC2aa0baaeaacqWGQbGAaeaacqaIYaGmaaGaeiikaGIaemiEaG3aaSbaaeaacqWGQbGAaeqaaiabcMcaPiabbsgaKjabdIha4naaBaaabaGaemOAaOgabeaaaeqabeGaey4kIipaaeaacqWGQbGAcqGH9aqpcqaIXaqmaeaacqWGWbaCaiabggHiLdaaaOGaeiOla4caaa@55E9@

The values of *S*_*i*_, *i *= 1, 2, ..., *p *can subsequently be plotted in a pie chart to indicate the sensitivity of each variable. Similarly, it is also possible to define sensitivity factors whose magnitudes indicate the importance of interaction effects [54,55].

#### Estimating worst-case settings

This section briefly discusses how the Bayesian emulator can be used to estimate settings of the inputs that give rise to maximum and minimum values of the output being modeled. A straightforward way to do this would be to directly maximize/minimize the posterior mean predicted by the emulator as a function of the input variables. This naive approach would work well provided the approximation quality of the emulator is sufficiently high. More general-purpose statistical criteria that employ the posterior variance along with the mean to enable the solution of complex optimization problems can be found in the engineering design literature [27].

In the present study, the probability of improvement (PoI) criterion was used to identify input settings that result in worst-case values of the output. The PoI criterion encapsulates the posterior mean as well as the variance (error bar) predicted by the emulator. For example, to find a geometry with maximum MWSS, the following optimization problem is solved.

**Maximise **: P[Y(**x**) > *y*^+^],

where *y*^+ ^denotes the highest value of MWSS among all the geometries in the training dataset used to construct the emulator. It can be noted that the PoI criterion indicates the probability that the output at a given point is greater than *y*^+^. Hence, by maximizing P[Y(**x**) > *y*^+^], it becomes possible to identify geometries that are likely to have higher values of MWSS compared to those in the training dataset.

Since the emulator prediction Y(**x**) at a given point **x **is a Gaussian random variable, the PoI can be computed exactly in terms of the standard normal distribution function. Note that for the case when it is desired to find geometries which have lowest MWSS, the criterion P[Y(**x**) <*y*^-^] can be maximized, where *y*^- ^denotes the lowest MWSS among all the geometries in the training dataset.

## Results and discussion

### Model geometry and problem description

**Figure 1:**
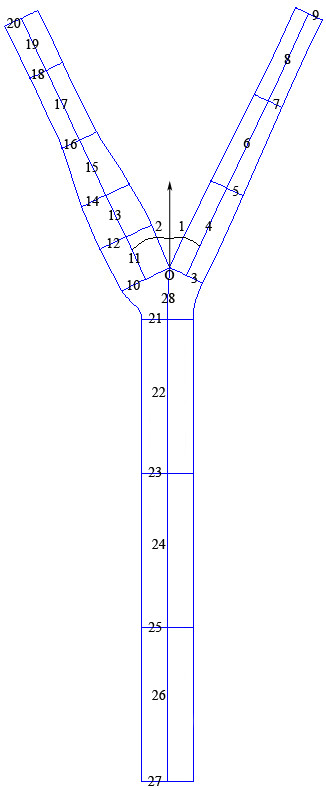
Diagrammatic representation of the human carotid artery bifurcation. Mean values (widths/lengths) at different locations are shown in Table 1. Note that the split ratio parameters are not shown here.

To demonstrate the applicability of the Bayesian Gaussian process modeling technique for assessing the relationships between a wide range of geometric parameters and MWSS, the widths at different cross-sections of the carotid artery (locations 3, 5, 7, 9, 10, 12, 14, 16, 18, 20, 21, 23, 25 and 27 in Figure 1) were considered as random variables which lead to a total of 14 input variables. The perturbation of each parameter was taken as 25% of the corresponding parameter's mean value given in Table 1. These perturbations were considered on each parameter keeping in mind the limitations posed by the geometry modeling tool and the mesh generator. To obtain a reasonable training dataset, 100 geometries were generated using optimized Latin hypercube sampling (LHS) technique [27,52]. LHS technique generates data that can be used to fit a Gaussian process model that reliably predicts the true trends of the input-output relationship.

All the geometries were created using CATIA [41] and the files were exported to GAMBIT [42] for mesh generation in standard for the exchange of product (STEP) model data format. The meshes thus generated were exported to FLUENT [43] where the numerical simulations were performed. The continuity equation

∂ui∂xi=0,
 MathType@MTEF@5@5@+=feaafiart1ev1aaatCvAUfKttLearuWrP9MDH5MBPbIqV92AaeXatLxBI9gBaebbnrfifHhDYfgasaacPC6xNi=xI8qiVKYPFjYdHaVhbbf9v8qqaqFr0xc9vqFj0dXdbba91qpepeI8k8fiI+fsY=rqGqVepae9pg0db9vqaiVgFr0xfr=xfr=xc9adbaqaaeGacaGaaiaabeqaaeqabiWaaaGcbaqcfa4aaSaaaeaacqGHciITcqWG1bqDdaWgaaqaaiabdMgaPbqabaaabaGaeyOaIyRaemiEaG3aaSbaaeaacqWGPbqAaeqaaaaakiabg2da9iabicdaWiabcYcaSaaa@384D@

and the laminar incompressible momentum equations

∂ui∂t+∂(ujui)∂xj=−1ρ∂p∂xi+ν∂2ui∂uj∂uj,
 MathType@MTEF@5@5@+=feaafiart1ev1aaatCvAUfKttLearuWrP9MDH5MBPbIqV92AaeXatLxBI9gBaebbnrfifHhDYfgasaacPC6xNi=xI8qiVKYPFjYdHaVhbbf9v8qqaqFr0xc9vqFj0dXdbba91qpepeI8k8fiI+fsY=rqGqVepae9pg0db9vqaiVgFr0xfr=xfr=xc9adbaqaaeGacaGaaiaabeqaaeqabiWaaaGcbaqcfa4aaSaaaeaacqGHciITcqWG1bqDdaWgaaqaaiabdMgaPbqabaaabaGaeyOaIyRaemiDaqhaaOGaey4kaSscfa4aaSaaaeaacqGHciITcqGGOaakcqWG1bqDdaWgaaqaaiabdQgaQbqabaGaemyDau3aaSbaaeaacqWGPbqAaeqaaiabcMcaPaqaaiabgkGi2kabdIha4naaBaaabaGaemOAaOgabeaaaaGccqGH9aqpcqGHsisljuaGdaWcaaqaaiabigdaXaqaaGGaciab=f8aYbaadaWcaaqaaiabgkGi2kabdchaWbqaaiabgkGi2kabdIha4naaBaaabaGaemyAaKgabeaaaaGccqGHRaWkcqWF9oGBjuaGdaWcaaqaaiabgkGi2oaaCaaabeqaaiabikdaYaaacqWG1bqDdaWgaaqaaiabdMgaPbqabaaabaGaeyOaIyRaemyDau3aaSbaaeaacqWGQbGAaeqaaiabgkGi2kabdwha1naaBaaabaGaemOAaOgabeaaaaGccqGGSaalaaa@6194@

were solved where *u*_*i*_, *ρ*, *p *and *ν *denote the velocity, density, pressure and the kinematic viscosity, respectively. A parabolic inflow velocity profile was applied at an average Reynolds number, ℜ = 271 (equivalent to the average Reynolds number of the pulse used in [56]), where

ℜ=ρU¯wμ
 MathType@MTEF@5@5@+=feaafiart1ev1aaatCvAUfKttLearuWrP9MDH5MBPbIqV92AaeXatLxBI9gBaebbnrfifHhDYfgasaacPC6xNi=xI8qiVKYPFjYdHaVhbbf9v8qqaqFr0xc9vqFj0dXdbba91qpepeI8k8fiI+fsY=rqGqVepae9pg0db9vqaiVgFr0xfr=xfr=xc9adbaqaaeGacaGaaiaabeqaaeqabiWaaaGcbaGaeyihHiSaeyypa0tcfa4aaSaaaeaaiiGacqWFbpGCcuWGvbqvgaqeaiabdEha3bqaaiab=X7aTbaaaaa@358A@

and density, *ρ *= 1035 kg.m^-3^, viscosity, *μ *= 0.0035 kg.m^-1^.s^-1^, the CCA diameter, *w *= 0.008 m and U¯
 MathType@MTEF@5@5@+=feaafiart1ev1aaatCvAUfKttLearuWrP9MDH5MBPbIqV92AaeXatLxBI9gBaebbnrfifHhDYfgasaacPC6xNi=xH8viVGI8Gi=hEeeu0xXdbba9frFj0xb9qqpG0dXdb9aspeI8k8fiI+fsY=rqGqVepae9pg0db9vqaiVgFr0xfr=xfr=xc9adbaqaaeGacaGaaiaabeqaaeqabiWaaaGcbaGafmyvauLbaebaaaa@2D1E@ is the mean velocity. The mass fluxes through the ICA and ECA were fixed in the ratio 70 : 30 (as used in the experimental studies conducted by [2]). The semi-implicit method for pressure-linked equations (SIMPLE) pressure correction algorithm was used with second order spatial accuracy. Other solver settings included a second order upwind scheme for momentum discretization; a standard scheme for the discretization of the pressure equation. The principal assumptions in the present study were to consider the flow as steady, blood as a Newtonian fluid and the walls to be rigid. After obtaining a converged solution, the magnitude of MWSS was extracted for each geometry in order to create the training data for constructing the emulator.

### Mesh dependence and flow setup

Before performing the numerical simulations, a mesh dependence study was performed on one of the candidate geometries from the training dataset and Table 2 summarizes the range of interval sizes, the overall cell count and the corresponding values of the magnitude of MWSS. It can be seen that the predictions for MWSS for cases with interval sizes 0.48 and 0.36 are close to each other. In order to find a trade-off between computational cost and accuracy, a fixed interval size of 0.48 was used on all the meshes which were subsequently generated on the candidate geometries. Structured mesh configurations were used for the common, internal and external carotid volumes. However, a hexcore mesh was used for the bifurcation volume which included tetrahedral elements on the outer edges of the volume and structured hexahedral elements in the center of the volume. The cell count was found to vary with respect to each geometry and on an average the number of cells used to discretize each geometry was about 110, 000.

**Table 2:** Mesh dependence configurations for a candidate geometry in the training data

*Interval Size*	*Cell Count*	*Magnitude of MWSS(Pa)*
0.96	18432	4.29743
0.84	23466	4.30954
0.72	35362	4.37759
0.60	53156	4.45912
0.48	115980	4.40611
0.36	241795	4.40634
0.24	835996	4.38985

### Influence of geometry

**Figure 2:**
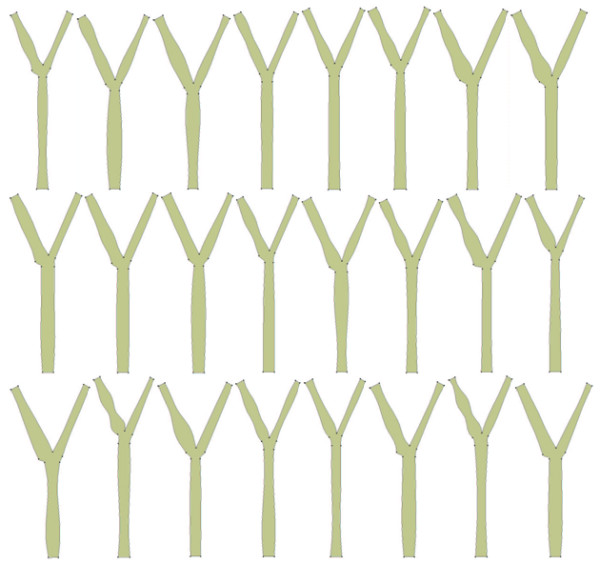
Different geometries obtained by varying the 14 parameters (24 geometries out of 100 are shown).

**Figure 3:**
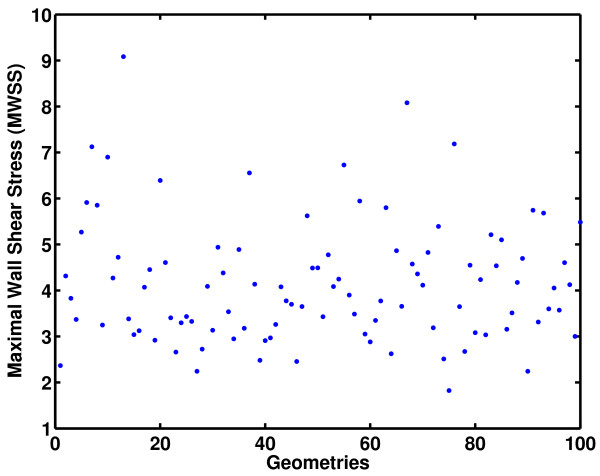
Scatter plot of the MWSS vs geometry cases considered (Units of MWSS in Pa).

Steady state simulations for the 100 geometries were undertaken using FLUENT [43] and MWSS for each geometry was extracted for constructing a Gaussian process model. It can be seen from Figure 2 that significant variations exist in the geometries considered for the present study. The objective is to construct an emulator that approximates the MWSS as a function of the geometry variables. The motivation for studying this relationship arises from the fact that elevated shear stress regions have the maximum probability of lesion formation. Further, MWSS enables the study of the role of high shear stresses in advanced occlusive lesions. The values of MWSS extracted for all the geometries that were used as training data for the emulator are shown in the form of a scatter plot in Figure 3. It can be seen from the figure that there is a significant change in the magnitude of MWSS for the cases considered for this study. This suggests that variations in geometry have a significant impact on the value of MWSS.

**Figure 4:**
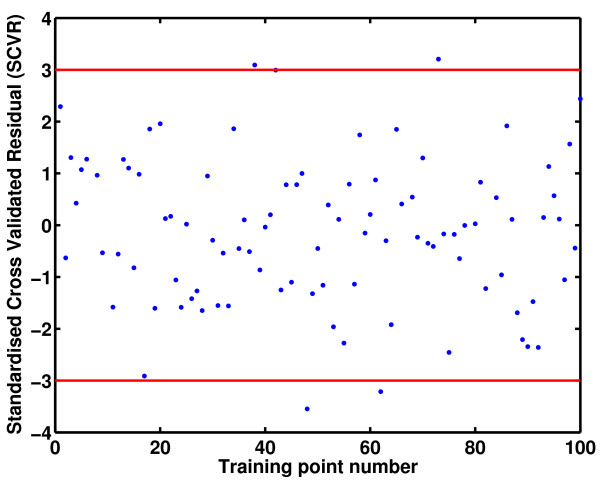
Standardized cross validated residual (SCVR) when 100 training points are used.

A Bayesian emulator was constructed using the 100 data points and the validation tests presented earlier were conducted to judge its predictive capability. Figure 4 shows the SCVR measure computed using the emulator. It can be noted that most of the values for SCVR lie within the interval [-3, 3], suggesting that the error bars predicted by the emulator are reasonably tight. It can be seen that a reasonably accurate model can be constructed in spite of the fact that a modestly sized training dataset was used.

### Distribution of maximal wall shear stress

**Figure 5:**
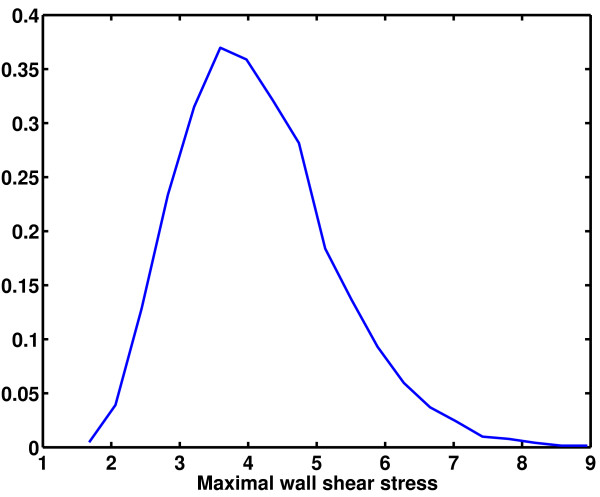
Probability density function of MWSS.

**Figure 6:**
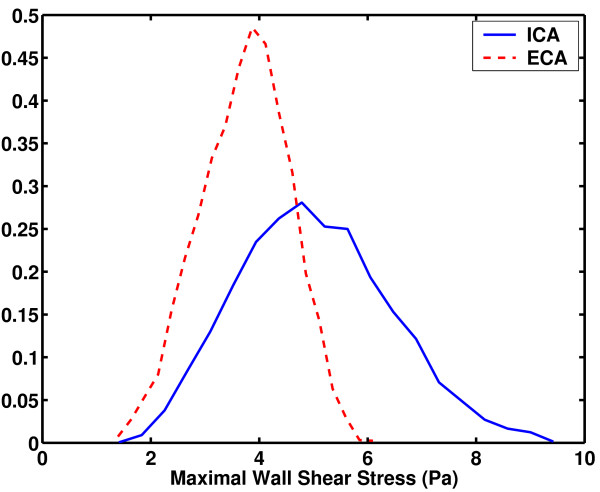
Probability density function of MWSS occurring on the ICA and ECA.

MCS with sample size of 5000 was applied to the Bayesian emulator to approximate the statistical moments of MWSS (Figure 5). It was observed that for some geometries, MWSS occurred on the inner wall of the ICA, whereas for others, it was located on the inner wall of the ECA. To investigate this issue in detail, the values of MWSS on the inner walls of the ICA and ECA were extracted to construct two separate emulators. Figure 6 shows the probability distribution of MWSS occurring on the ICA and ECA, respectively. These distributions were generated using MCS applied to the emulators with sample size 5000. The probability of MWSS occurring on the inner wall of the ICA was also calculated using the emulator and it was found to be approximately 0.72. This suggests that for most of the geometries, the MWSS occurs on the inner wall of the ICA.

### Main effects

**Figure 7:**
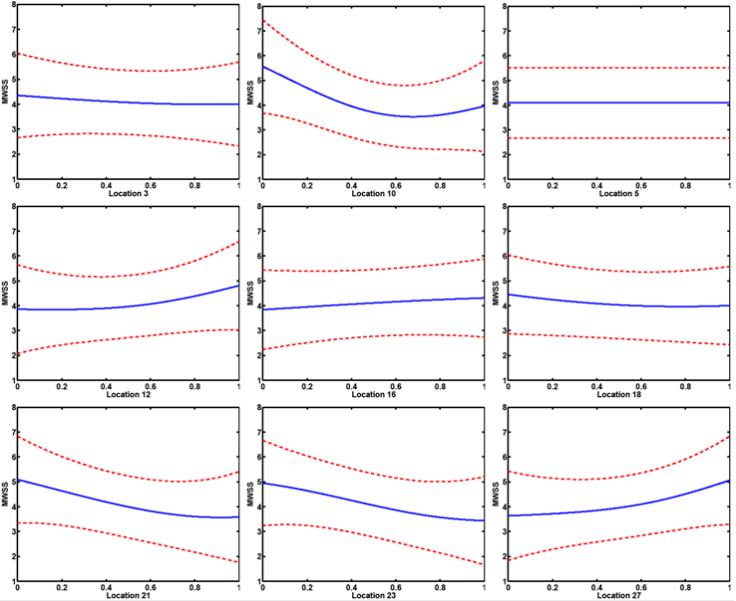
Main effect plots of the parameters (9 out of 14) are shown in which the middle line is the estimated effect and the other lines are the ± 95% confidence limits based on the standard error. On the x-axis of each subplot, the widths at the locations are normalized using their bounds and on the y-axis of each subplot, MWSS (units – Pa) is shown. Note that the main effects of the remaining parameters at locations 7, 9, 14, 20 and 25 (not shown) are flat and similar to the effect at location 5 shown in this figure.

Main effect plots generated by integrating out the other covariates are shown in Figure 7. The main effect plots of the widths at locations 5, 7, 9, 14, 20 and 25 were very flat. In contrast, the main effect plots for the widths close to the flow divider at locations 3, 10 and 21 were nonlinear. Furthermore, the parameter at location 12 governing the width of the sinus bulb also exhibited a nonlinear behavior. It has already been observed that the bifurcation region is vulnerable to atherogenesis due to the nature of its geometry [11]. Furthermore, high shear regions were identified on the inner walls of the ICA and ECA. From the present study, it can be inferred that the probability of plaque rupture, plaque ulceration and thromboembolism is maximum near the bifurcation region, particularly on the inner walls of the ICA and ECA. In particular, we infer that geometries having larger curvature of the sinus bulb tend to have high values of MWSS. Also, a significant nonlinear behavior was observed at location 27 which corresponds to the inlet diameter at the upstream of the CCA. This is due to the fact that at a constant Reynolds number, changes in inlet area influences the magnitude of the velocity which consequently determines the shear stress distribution downstream.

### Probability of improvement

**Figure 8:**
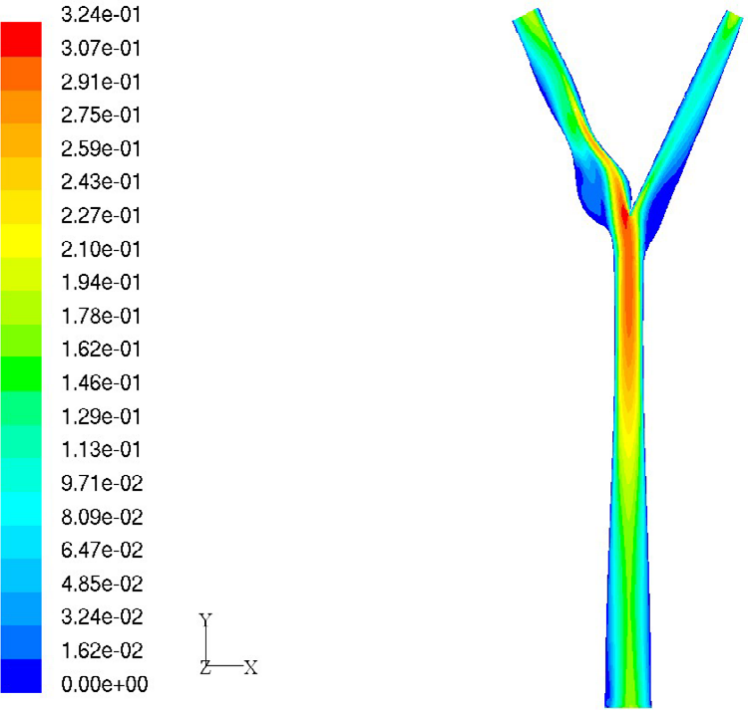
Geometry having maximum value of MWSS. Velocity magnitude (m/s) contours on center plane.

We now discuss the application of Bayesian Gaussian process modeling to predict new geometries which have highest and lowest MWSS. The basic idea is to use the probability of improvement (PoI) criterion for optimization. The motivation is to employ these geometries and their corresponding values of MWSS for defining metrics which perhaps can be further used for assessment of stroke risk. An optimization study was conducted to predict a geometry which has the highest MWSS using the PoI criterion. Subsequently, the true value of the MWSS was computed using FLUENT [43]. Note that, if the CFD prediction does not agree with the emulator prediction, then a new emulator can be constructed by appending the point to the original training dataset and the criterion in Equation (18) can be maximized in order to obtain a new geometry. This update procedure can be carried out in an iterative fashion until a favorable geometry is obtained. However, the CFD predicted value of MWSS for this case (9.2879 Pa) was found to agree closely with the emulator prediction (9.1493 Pa). It can be seen that the CFD predicted value of the MWSS was greater than the maximum value of MWSS in the training dataset (9.0841 Pa). Figure 8 shows the velocity contours and more importantly the shape of the predicted arterial geometry. It can be clearly seen that the shape of the predicted arterial geometry has a sinus bulb that has larger curvature compared to the baseline case.

**Figure 9:**
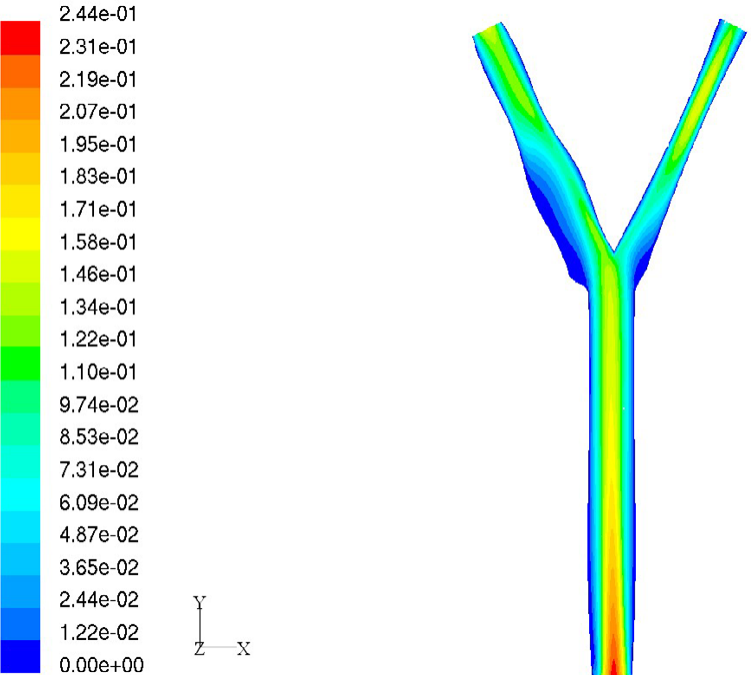
Geometry having minimum value of MWSS. Velocity magnitude (m/s) contours on center plane.

Similarly, the criterion P[Y(**x**) <*y*^-^] was maximized to obtain a geometry which has lowest MWSS. In this case, it was found that the CFD prediction (2.1464 Pa) was not in close agreement with the emulator prediction (1.3539 Pa). Hence, the update procedure was repeated four times and it was found that the CFD predicted value of the MWSS (1.7907 Pa) for the new geometry was less than the minimum value of MWSS among the training dataset (1.8233 Pa). Figure 9 shows the velocity contours and the shape of the arterial geometry for this case. The results obtained for these cases suggest the applicability of the emulator model to efficiently predict arterial geometries having low and high values of MWSS by optimizing a suitable objective function.

### Unsteady simulations

So far, the applicability of Bayesian modeling for statistical analysis has been demonstrated by performing steady state simulations on the candidate geometries. In this section, unsteady simulations were performed on the same candidate geometries and the PoI criterion was applied. As an inflow boundary condition at the inlet of the CCA, we used a mean inflow pulse taken from [56]. A time dependence study was performed on the mesh interval size 0.48 (mesh size taken for the steady state flow analysis) with time steps 0.001, 0.0001 and 0.00001 seconds. Since the flow is pulsatile, we computed the maximum value of the average wall shear stress in a time period (T = 0.917 sec) for each case. Henceforth, this new metric is denoted as maximal average shear stress (MASS). The MASS values for these cases were found to be 4.1405, 4.1347 and 4.1057 Pa, respectively. Consequently, 0.0001 sec was selected providing a reasonable balance between accuracy and computational cost. All unsteady simulations employed the pressure implicit with splitting of operators (PISO) pressure-velocity coupling scheme in FLUENT [43]. For the purpose of validation, the average wall shear stress for the case with mesh interval size 0.48 mm and time step 0.0001 sec was computed and found to be 0.692 Pa. This value lies within the accepted range of values reported in [57]. Recall that the dimensions and boundary conditions of the baseline case closely match with the data described in [2,6,40,56].

**Figure 10:**
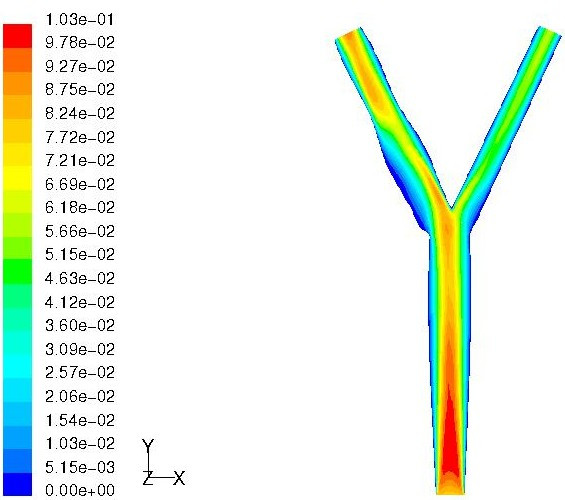
Geometry having minimum value of MASS. Velocity magnitude (m/s) contours on center plane.

After pulsatile simulations were performed on the same candidate geometries, an emulator was constructed and the PoI criterion was used to predict an arterial geometry having a low value of MASS. The contours of velocity and wall shear stress at the end of the cycle for this geometry are shown in Figure 10. By closer inspection of Figures 9 and 10, it can be seen that the shapes of the two geometries resemble each other very closely. As a consequence, we infer from the perspective of Bayesian modeling that steady state simulations can accurately identify key geometric parameters which influence MWSS in the carotid bifurcation at a fraction of the computational cost of pulsatile simulations. For example, each pulsatile simulation takes about 14.5 CPU hours on an AMD Athlon XP 2800+ processor [58] whereas a steady state simulation for the same case takes only about 24 minutes.

### Potential diagnostic indicators

So far, we demonstrated the ability of the Bayesian Gaussian process model to predict geometries which have highest and lowest values of MWSS. We now discuss how the optimization results can be employed in a clinical scenario to estimate the degree of severity with respect to MWSS for an individual patient. For example, given the geometry of the carotid bifurcation of a patient using an MRI scan [36,38], a least squares fit to the proposed CAD representation can be first carried out. The parameters from the fit can then be fed into the emulator to approximate the MWSS (or any other metric of interest) and its corresponding error bars in real time. The resulting approximation for the MWSS can then be compared with the values obtained for the geometries in the earlier section. In particular, if Γ^m
 MathType@MTEF@5@5@+=feaafiart1ev1aaatCvAUfKttLearuWrP9MDH5MBPbIqV92AaeXatLxBI9gBaebbnrfifHhDYfgasaacPC6xNi=xH8viVGI8Gi=hEeeu0xXdbba9frFj0xb9qqpG0dXdb9aspeI8k8fiI+fsY=rqGqVepae9pg0db9vqaiVgFr0xfr=xfr=xc9adbaqaaeGacaGaaiaabeqaaeqabiWaaaGcbaGafu4KdCKbaKaadaWgaaWcbaGaeeyBa0gabeaaaaa@2ED8@ denotes the approximation to the MWSS for a given geometry, Γ_g _is the minimal value of MWSS and Γ_b _is the maximum value of MWSS, then the degree of severity for this case with respect to the metric MWSS can be defined as

Ds=(Γ^m−Γg)(Γb−Γg).
 MathType@MTEF@5@5@+=feaafiart1ev1aaatCvAUfKttLearuWrP9MDH5MBPbIqV92AaeXatLxBI9gBaebbnrfifHhDYfgasaacPC6xNi=xI8qiVKYPFjYdHaVhbbf9v8qqaqFr0xc9vqFj0dXdbba91qpepeI8k8fiI+fsY=rqGqVepae9pg0db9vqaiVgFr0xfr=xfr=xc9adbaqaaeGacaGaaiaabeqaaeqabiWaaaGcbaWenfgDOvwBHrxAJfwnHbqeg0uy0HwzTfgDPnwy1aaceaGae83aXt0aaSbaaSqaaiabdohaZbqabaGccqGH9aqpjuaGdaWcaaqaamaabmaabaGafu4KdCKbaKaadaWgaaqaaiabb2gaTbqabaGaeyOeI0Iaeu4KdC0aaSbaaeaacqqGNbWzaeqaaaGaayjkaiaawMcaaaqaamaabmaabaGaeu4KdC0aaSbaaeaacqqGIbGyaeqaaiabgkHiTiabfo5ahnaaBaaabaGaee4zaCgabeaaaiaawIcacaGLPaaaaaGaeiOla4caaa@4C3E@

Alternatively, the geometry of the patient can be compared with those predicted in the previous section. A similar approach can be applied for evaluating the bounds with respect to any indicator which synthesizes different flow behavior from which D
 MathType@MTEF@5@5@+=feaafiart1ev1aaatCvAUfKttLearuWrP9MDH5MBPbIqV92AaeXatLxBI9gBaebbnrfifHhDYfgasaacPC6xNi=xH8viVGI8Gi=hEeeu0xXdbba9frFj0xb9qqpG0dXdb9aspeI8k8fiI+fsY=rqGqVepae9pg0db9vqaiVgFr0xfr=xfr=xc9adbaqaaeGacaGaaiaabeqaaeqabiWaaaGcbaWenfgDOvwBHrxAJfwnHbqeg0uy0HwzTfgDPnwy1aaceaGae83aXteaaa@374D@_*s *_can be estimated. It should be noted that Γ_g _and Γ_b _have been estimated in a laminar flow regime with fixed outflow boundary conditions and by assuming the walls to be rigid. To extend this concept of assessment on the severity of disease for a more physiologically realistic case, we can incorporate realistic boundary conditions and the arterial wall distensibility to estimate D
 MathType@MTEF@5@5@+=feaafiart1ev1aaatCvAUfKttLearuWrP9MDH5MBPbIqV92AaeXatLxBI9gBaebbnrfifHhDYfgasaacPC6xNi=xH8viVGI8Gi=hEeeu0xXdbba9frFj0xb9qqpG0dXdb9aspeI8k8fiI+fsY=rqGqVepae9pg0db9vqaiVgFr0xfr=xfr=xc9adbaqaaeGacaGaaiaabeqaaeqabiWaaaGcbaWenfgDOvwBHrxAJfwnHbqeg0uy0HwzTfgDPnwy1aaceaGae83aXteaaa@374D@_*s*_. Furthermore, offline simulations on an extended set of geometries can be carried out to improve the predictivity of the emulator.

## Conclusion

This paper presented a computer-based Bayesian modeling approach for data mining. The methodology was applied on a hemodynamic problem in which the geometric parameters affecting maximal wall shear stress (MWSS) in the human carotid bifurcation were analyzed. A design of experiments approach was employed to generate a set of geometries for numerical simulations using a Navier-Stokes solver. The results obtained from these runs were then used as training data to construct a Bayesian Gaussian process model. Applications of the resulting emulator include (i) uncertainty quantification when the inputs were modeled as random variables, (ii) the quantification of the sensitivity of an output quantity of interest with respect to the input parameters, and (iii) the ability of the model to predict geometries having low and high values of maximal wall shear stress using the probability of improvement criterion which can be subsequently used for estimating degree of severity with respected to any patient.

The work in this paper seeked to directly compare the effect of varying the geometric parameters on the maximal wall shear stress for the human carotid bifurcation. However, this metric only highlights the role of elevated shear regions. Using the same principle, new metrics can be used to correlate the regions of disturbed flow and sites of arterial disease [31-34,59]. The main focus here was to demonstrate the applicability of the Bayesian modeling approach to mine data generated from hemodynamic simulations. Physiologically more relevant results can be obtained by considering the fluid-wall interactions and non-Newtonian nature of blood along with more realistic boundary conditions. More importantly, this analysis can be carried out on any arterial location that is vulnerable to hemodynamic derangements. Furthermore, new parameters can be introduced into the CAD model that can simulate the presence of stents or grafts. Subsequently, optimization studies can be performed on these cases that can improve the design and control of these artificially implanted devices [60-66]. We hope that the Bayesian Gaussian process modeling methodology can be efficiently integrated with well known image reconstruction techniques to generate a powerful paradigm of designing, optimizing and controling patient-specific blood flow behavior.

## Competing interests

The author(s) declare that they have no competing interests.

## Appendix A

### Construction of the parameterized CAD model

**Figure 11:**
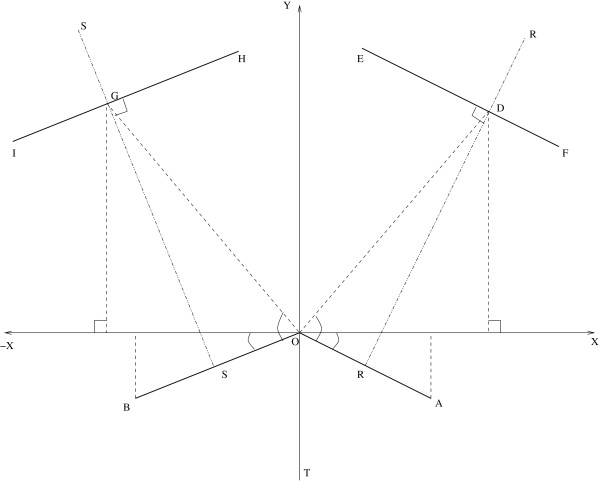
Carotid artery geometry construction. OA and OB correspond to locations 3 and 10 in Figure 1 whereas EF and HI correspond to other widths in the ECA and ICA respectively.

The parametric CAD definition for the Y-shaped model used the junction between the inner walls of the ICA and ECA as the origin and starting point of the construction. Figure 1 depicts the complete definition. With reference to Figure 11, first, lines OA and OB were drawn from the origin, O, for the ECA and ICA, respectively, that define the orientations and the respective widths at the bifurcation. Second, a perpendicular bisector, RR, to the ECA was used to specify a number of downstream sections (e1 to e3 corresponding to the locations 5, 7 and 9 in Figure 1) each of which possesses three parameters: a distance from OA, d; a width, w and; a split ratio, r, defining the relative position on RR. The endpoints of each section were then joined by splines to define the inner and outer ECA edges. Third, a similar procedure was followed for the ICA using a perpendicular bisector, SS, and sections (i1 to i5 corresponding to the locations 12, 14, 16, 18 and 20 in Figure 1). Fourth, a line, OT, was drawn down from O, parallel to the y-axis, and the CCA was defined by sections (c0 to c3 corresponding to the locations 21, 23, 25 and 27 in Figure 1) specified by their distances from O, widths and split ratios. It should be noted here that the lines referred to in the description are only for construction purposes since it is the coordinates of the end-points of each section that were needed through which splines were drawn. subscript 'e' refers to the ECA). The split ratios were all assumed equal to unity for the ECA and ICA sections and equal to 1.2 for the CCA section. Once the coordinates of all the points were evaluated, splines were connected between them with tangency constraints applied at each join. The geometry thus generated is a two dimensional parametric representation of a human carotid artery. To begin the transformation from 2D into 3D, semi-circles were added to the baseline geometry at the top and bottom of the CCA, ICA and the ECA in order to facilitate lofting of the outer surfaces. These semi-circles were defined parametrically to allow the model to automatically update when changes are made to the geometry definition. The artery wall surfaces were then created by lofting elements of the semi-circles along the edge boundaries for each artery wall. Additional splines were added where appropriate to assist the lofting of each surface. Constructed in this way, parameters of the Y-shaped model can be simply manipulated to control the complete shape. More details of the parameterized CAD model can be found in [33].
